# Repurposing of known drugs for COVID-19 using molecular docking and simulation analysis

**DOI:** 10.6026/97320630019149

**Published:** 2023-02-28

**Authors:** Piyush Bhanu1, Anagha S Setlur, Chandrashekar K, Vidya Niranjan, Nisha Hemandhar Kumar, Sakshi Buchke, Jitendra Kumar, Anita Rani, Sushil M Tiwari, Vachaspati Mishra

**Affiliations:** 1 Xome Life Sciences, Bangalore Bio Innovation Centre (BBC), Helix Biotech Park, Bengaluru, Karnataka- 560100, India; 2 Department of Biotechnology, RV College of Engineering, RV Vidyanikethan Post, Mysuru Road, Bengaluru 560059, India; 3 Department of Biotechnology, RV College of Engineering, RV Vidyanikethan Post, Mysuru Road, Bengaluru 560059, India; 4 Department of Biotechnology, RV College of Engineering, RV Vidyanikethan Post, Mysuru Road, Bengaluru 560059, India; 5 Institute of Neuro and Sensory Physiology, University Medical Centre, Goettiengen - 37075, Germany; 6 Xome Life Sciences, Bangalore Bio Innovation Centre (BBC), Helix Biotech Park, Bengaluru, Karnataka- 560100, India; 7 Bangalore Bio Innovation Centre (BBC), Helix Biotech Park, Electronics City Phase- 1, Bengaluru-560100, Karnataka, India; 8 Department of Botany, Dyal Singh College, University of Delhi, New Delhi 110003, India; 9 Department of Botany, Hansraj College, University of Delhi, Delhi 110007, India; 10 Department of Botany, Deen Dayal Upadhyay College, University of Delhi, Delhi 110078, India

**Keywords:** SARS-CoV-2, molecular dynamics simulation, receptor, ligand, COVID-19

## Abstract

We selected fifty one drugs already known for their potential disease treatment roles in various studies and subjected to docking and molecular docking simulation (MDS) analyses. Five of them showed promising features that are discussed and suggested as potential candidates for repurposing for COVID-19. These top five compounds were boswellic acid, pimecrolimus, GYY-4137, BMS-345541 and triamcinolone hexacetonide that interacted with the chosen receptors 1R42, 4G3D, 6VW1, 6VXX and 7MEQ, respectively with binding energies of -9.2 kcal/mol, -9.1 kcal/mol, -10.3 kcal/mol, -10.1 kcal/mol and -8.7 kcal/mol, respectively. The MDS studies for the top 5 best complexes revealed binding features for the chosen receptor, human NF-kappa B transcription factor as an important drug target in COVID-19-based drug development strategies.

## Background:

We previously showed that Sulindac, a non-steroidal (NSAID) drug that interacted with a key human transcription factor NFkB leading to its suppression that might have the potential to significantly control COVID-19, which needs to be ascertained through clinical studies [1]. Our current work emphasizes on 51 selected drugs that are chosen from the list of existing drugs as effective treatment options for various human diseases and these were also considered currently based on their in silico properties as performed in the current work. Several strategies to develop drugs and vaccines to control SARS-CoV-2 and treat COVID-19 were attempted since emergence of this disease, but this disease recurs and still exists. The major problems faced by scientists in combating COVID-19, have been the occurrence of viral complementary proteins in the hosts that are required for multiplication and life-cycle completion of the virus. The expressions of these proteins possibly need to be manipulated in order to control COVID-19 disease symptoms. Hence, it is important that protein-protein interactions between viral proteins and their host cellular proteins and associated cofactors be identified so that an efficient mechanism to understand the virus can be found out and potential drug targets can be identified [2]. There are some significant reports describing this approach in receptor identification that uses different strategies, such as affinity purification (AP) [3, 4, 5, 6, 7, 8, 9, 10] and proximity labelling-based strategies [11, 2]. Furthermore, suggestions have emerged stating that SARS-CoV-2 proteins and some other protein domains are important to the viral lifecycle [12]. Domains in proteins are crucial in being used as the functional units connected through signalling networks within a cell to the target [13, 14]. Additionally, motifs are amino acid sequences that are used for viral interaction with the host proteins within the host cells [15]. Motifs are employed by the viruses to mimic and hijack the host cell's essential process for its own survival [16]. Further detailed information on domain-motif interactions are available [17] that offers some valuable clue in conducting further bioinformatic studies related to drug discovery. In the current scenario, constructing a downstream network including all potential viral receptors, host cell proteases, and cofactors is necessary and should be used as an additional criterion for the validation of critical host machinery used for COVID-19 viral processing for therapeutic intervention. Liu *et al.* [18] have worked on similar lines and have come out with one significant drug named as methotrexate that has been immensely useful in drug development program. Due to the high viral mutation rates, drugs resistant to viruses can occur that leads to treatment failure, especially for infections caused by RNA viruses [19]. In contrast, host-targeting drugs can avoid such effects because of the low evolutionary divergence of host proteins. Therefore, as outlined above, it is necessary to construct a comprehensive virus-host proteome interaction atlas that can be used to identify the cellular functions that are mandatory for viral processing and, in turn, to develop effective therapeutic strategies against SARS-CoV-2 and new emergent strains. Our current work targets host proteome, i.e., human transcription factor, such as NF-κB primarily and other associated interactome, while identifying unique interaction parameters employing molecular dynamics simulation and other accessory tools that could generate supporting data for the repurposing of the presently studied molecules for COVID-19 disease control post thorough clinical trials.

## Materials and methods:

## Retrieval of the proteins and ligand structures:

The protein structures for five chosen receptors (1R42, 4G3D, 6VW1, 6VXX and 7MEQ) were retrieved in .pdb format from RCSB PDB (Research Collaboratory for Structural Bioinformatics- Protein Databank) [20]. All receptors were downloaded in high resolution. The receptors downloaded were all structures analysed via X-ray diffraction with a resolution greater than 1.5 Å and less than 3 Å. 1R42 had a resolution of 2.2 Å, 4G3D had a resolution of 2.9 Å, 6VW1 of 2.68 Å, 6VXX of 2.8 Å and 7MEQ, a resolution of 1.95 Å. Fifty-one ligands belonging to various classes such as IKK complex inhibitors, IκB degradation inhibitors, NF-κB nuclear translocation inhibitors, p65 acetylation inhibitors, NF-κB DNA binding inhibitors, NF-κB transactivation inhibitors, p53 inductors and NF-κB activators and inducers were retrieved from NCBI PubChem [21, 22] and ZINC databases [23]. These were saved in .sdf format which were later converted to appropriate docking format prior to molecular docking studies. Some ligands that did not have any available 3D structures for direct download from the small molecule databases, the canonical SMILES from PubChem database were converted to 3D structures and these were downloaded in .sdf format. The 3D structures were generated using CORINA [24].

## Virtual screening:

Virtual screening for 51 ligands against 5 selected major protein targets that play a role in the infection of SARS-CoV-2 was carried out to find the top 5 best docked complexes, which were then taken forward for molecular simulation studies.

## Molecular docking studies and visualization of best docked complexes:

All the 51 ligands were initially converted from .sdf to pdbqt format using Autodock version 4.2.6 and using JSME for molecular editing where ever necessary [25] along with Open Babel Tool [26] to bring the ligands to appropriate docking format. The five proteins were first loaded into PyRx [27], virtual screening software for computational drug discovery. PyRx is open-source software that has an interactive user-interface and runs efficiently on all important operating systems such as Windows, Linux and Mac [27]. It provides a graphical user interface for setting up the dockings via AutoDock and AutoDock Vina and also to analyze the results relatively easily.

The .pdb files of both the protein targets and the 51 ligands were prepared by converting to .pdbqt format. The molecules were then selected for multiple ligands with single protein docking. All 51 ligands and the protein of interest were selected for molecular docking. The grid box was adjusted to cover the whole protein molecule so that the ligands can search for the binding sites in the entire protein. The x, y, and z coordinates were noted after adjusting the grid box for each protein. The exhaustiveness was pre-set to 8 for accomplishing thorough docking and for obtaining accurate binding energies between the protein-ligand complexes. Binding energy was calculated as the sum total of all intermolecular interactions between the ligand and the proteins.

Binding energy: a X vdW + b X Coul + Hbond + Metal + Lipo + BuryP + RotB + Site

Where, 'a' and 'b' are co-efficient constants for vdW and Coul respectively, vdW = Vander Waals energy, Coul = Coulomb's energy, Metal = binding with metal ions, Hbond = Receptor hydrogen bonding, Lipo = Lipophilic interactions, BuryP = buried polar group penalty, Site = polar interactions active sites, RotB = rotatable bond penalty.

PyRx provides the top 9 best docked conformers with varying RMSD values of the docked complexes. The conformer having the best RMSD value and the least binding energy was selected for visualization in order to examine the interactions at the molecular level. Furthermore, the docked complexes with the best binding energies were visualized in 2D and 3D. The 2D visualization was carried out in Maestro workspace of Desmond software [28]. The .pdbqt output files for the protein and ligand were first opened in PyMol [28]. The complex was saved and exported as .pdb and then loaded onto the Maestro workspace for viewing ligand interactions in 2D. The amino acid interactions between the receptor and ligand were noted in terms of hydrogen bond interactions and hydrophobic interactions. The protein-ligand interactions in 3D were explored using the .pdb complex exported from PyMol by loading it in DisCoVery Studio [29].

## Molecular dynamic simulations:

The protein stability upon ligands bound to them, was scored at 100ns employing simulation studies by the use of Desmond [28] for the top 5 best docked ligands and their proteins because of the valuable features of Desmond [30, 31, 32]. A simulation study for 100ns is considered standard for docked complexes to attain a state of equilibration. Before the molecules were loaded into the Maestro workspace after setting the working directory, the protein pre-processing was performed. The pre-processing of proteins was on the interaction complexes using various parameters such as examining for potential errors in the structures, minimization of the structures for 500 steps via steepest decent algorithm, and removal of water molecules was performed. The simulation environment was assembled by utilizing system builder in the Maestro GUI. The boundary conditions were defined by the orthorhombic box of minimized volume that compressed the complex by 5 Å on axis. The force field used was OPLS3e and the system was neutralized by addition of Na+ or Cl- ions. Neutral pH of 7.0 was set for simulations to impart precise states of protonation for the residues at specific pH and simulation criteria.

While performing the simulation for 100ns, the top 5 interaction complexes were imported one by one into the maestro workspace. The trajectory recording interval was set to 0.1 ns. Normal Volume Temperature (NVT) was used for simulation at 310K. Simulation was carried out generally using the following steps: Brownian dynamics NVT, where T=10K, Brownian dynamics NVT, where T=50K, NPT (Normal Pressure Temperature), where T=50K, NVT production with constraints removed for 200ps, NVT production with removal of constraints for 500ps and the ultimate simulation for 200ps with a temperature of 310K and timestep of 2 femto-seconds (fs). A simulation interaction diagram tool was employed for examining the results inclusive of RMSD value fluctuations as well as the protein-ligand contacts. For each docking performed, the RMSD (root mean square deviation) was computed. RMSD values measured the average change in the displacement of a particular selection of atoms for a specified frame with regards to a frame of reference [28].

 RMSD was calculated for all existing frames in the trajectory and is given as:(see PDF)

Here, N is the overall number of atoms in the atom selection; tref is time of reference (the first frame is the reference and is considered as time t=0); r' indicates the chosen atoms' positions within the frame x after superimposition on the frame of reference, where x is recorded at tx time [10]. This procedure was iterated for every frame in the simulation trajectory to achieve the 100ns simulation dynamics. Protein RMSF, ligand RMSF and the protein-ligand contacts were also scrutinized to comprehend the stability of the docked complex and its interactions at the molecular level.[Table 1]

##  Results:

The five selected receptors and the 51 shortlisted ligands were successfully retrieved and prepared for molecular docking studies.

## Comprehension of binding efficacies between drug and receptor via molecular docking:

The experimental small molecules were docked against the five receptors and negative binding energies were observed for all the interaction complexes. The results showed that boswellic acid bound maximum to receptor 1R42 (Native Human Angiotensin Converting Enzyme-Related Carboxypeptidase) with a binding energy of -9.2k cal/mol. This was followed by Pimecrolimus (binding energy -9 kcal/mol) and Tacrolimus (binding energy -9 kcal/mol). Similarly, Pimecrolimus bound strongest with receptor 4G3D (human NF-kappaB inducing kinase) with an energy of -9.1 kcal/mol, followed by tacrolimus (-9.1 kcal/mol) and GYY_4137 (-8.9 kcal/mol). GYY_4137 bound strongly with 6VW1 (SARS-CoV-2 chimeric receptor-binding domain complexed with its receptor human ACE2) with a docking score of -10.3 kcal/mol, followed by triamcinolone acetonide and triamcinolone hexacetonide with energies -10.2 kcal/mol and -9.9 kcal/mol, respectively. Similarly, ligand BMS_345541 bound to receptor 6VXX (SARS-CoV-2 spike glycoprotein) with a score of -10.1 kcal/mol, closely ensued by GYY-4137 (binding energy -9.9 kcal/mol) and boswellic acid (binding energy -9.7k cal/mol). Triamcinolone hexacetonide had a binding energy of -8.7 kcal/mol when bound to the 7MEQ receptor (human TMPRSS2 in complex with Nafamostat). GYY_4137 and mesalamine also bound well with 7MEQ at -8.7 kcal/mol and -8.2 kcal/mol, respectively. Detailed docking scores and top 5 best ligands against each receptor is provided in Table 3. Next, the top five interaction complexes were taken forward for visualization studies.

##  Visualization of the docked complexes and amino acid interaction studies:

A 3D visualization of the top 5 docked complexes for each receptor revealed several amino acid interactions that had undergone hydrogen and hydrophobic interactions with each other. Interaction complex boswellic acid and 1R42 showed hydrogen bonds for residues Arg273 (A), Asn277 (A) belonging to the A chain, while residues Trp271 (A), Phe274 (A) and Phe504 (A) demonstrated hydrophobic interactions. Complex pimecrolimus-4G3D evidenced hydrogen bonds at residues Ala350 (A), Thr383 (A) and Glu384 (A) of the A chain of the protein and amino acid residues Pro454 (A), Tyr456 (A), Lys482 (B) and Thr597 (B) showed hydrophobic interactions. Interacting complex 6VW1-GYY_4137 had hydrogen bonds at Glu406 (B) and His345 (B) of the B chain, and hydrophobic associations with Phe274 (B), Thr371 (B) and Phe504 (B). Likewise, 6VXX-BMS_345541 complex was observed to have hydrogen bonds at Asn1023 (B) and Arg1019 (B) belonging to the B chain of the receptor and hydrophobic interactions with Ala1020 (B), Ala1020 (C), Leu1024 (A) and Leu1024 (C). 7MEQ-Triamcinolone_hexacetonide showed hydrogen bonds at Asp451 (A), Ser448 (A), Glu260 (A), Trp267 (A) and Leu263 (A) and hydrophobic associations at Glu260 (A), Trp267 (A), Trp380 (A) and Trp453 (A). Amino acid interaction studies revealed that key residues of the selected receptors play a major role in the mechanism of interaction during the binding events. Detailed amino acid interactions of the top five complexes for each receptor are provided in Table 2.

**Figure 1:**
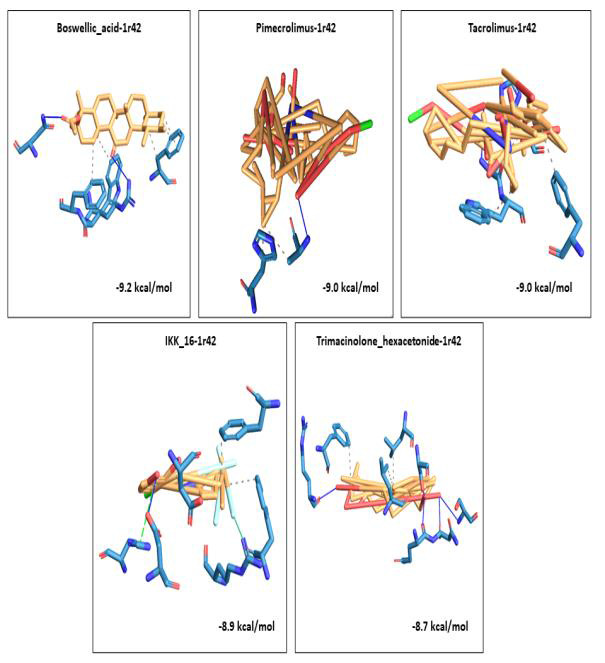
3D visualization of the top five best docked complexes for receptor 1R42. Their binding energies and the amino acid interactions between the ligands and the receptor, as viewed and analysed in PLIP tool. Orange colour represented the ligand part of the complex, while the bluish colours indicated the areas of the proteins to which the ligands interacted.

**Figure 2:**
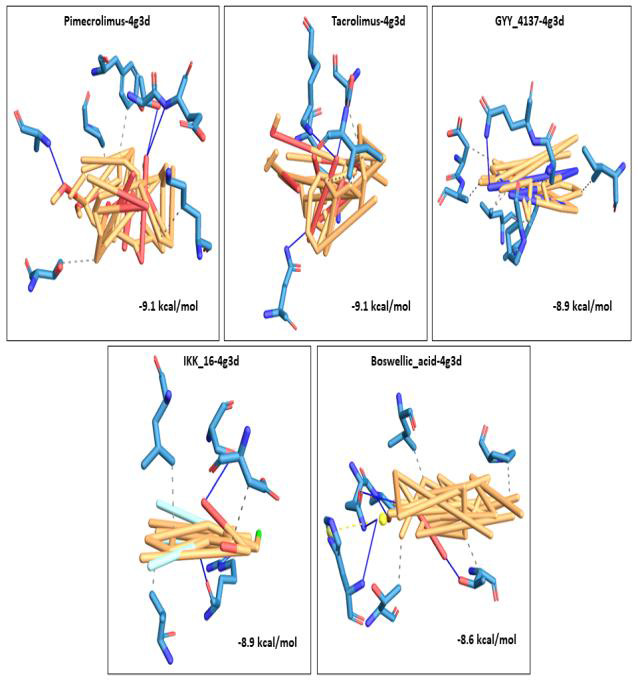
3D visualization of the top five best docked complexes for receptor 4G3D. Their binding energies and the amino acid interactions between the ligands and the receptor, as viewed and analysed in PLIP tool. Orange colour represented the ligand part of the complex, while the bluish colours indicated the areas of the proteins to which the ligands interacted.

**Figure 3:**
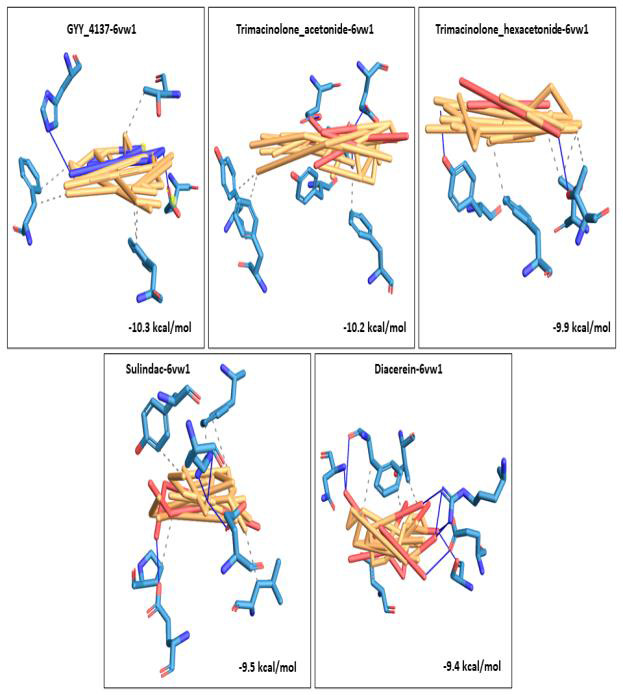
3D visualization of the top five best docked complexes for receptor 6VW1. Their binding energies and the amino acid interactions between the ligands and the receptor, as viewed and analysed in PLIP tool. Orange colour represented the ligand part of the complex, while the bluish colours indicated the areas of the proteins to which the ligands interacted.

**Figure 4:**
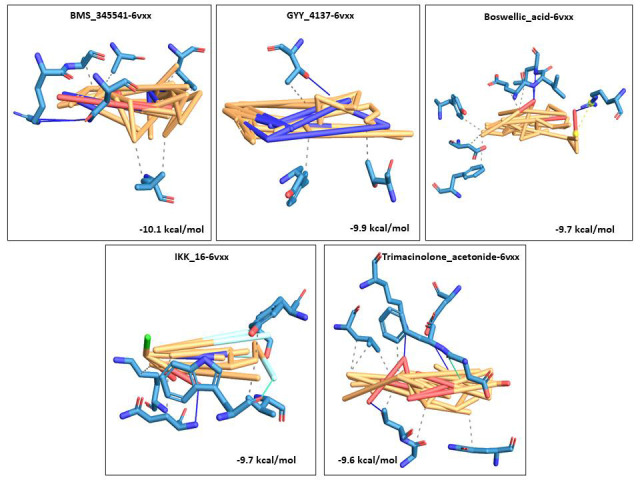
3D visualization of the top five best docked complexes for receptor 6VXX. Their binding energies and the amino acid interactions between the ligands and the receptor, as viewed and analyzed in PLIP tool. Orange colour represented the ligand part of the complex, while the bluish colours indicated the areas of the proteins to which the ligands interacted.

**Figure 5:**
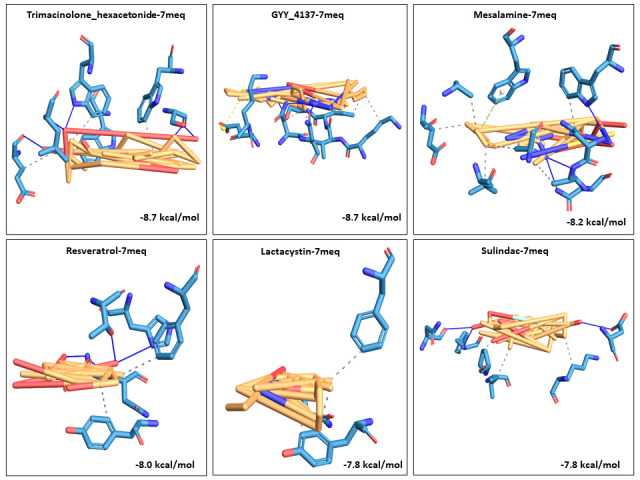
3D visualization of the top five best docked complexes for receptor 7MEQ. Their binding energies and the amino acid interactions between the ligands and the receptor, as viewed and analysed in PLIP tool were shown here. Orange colour represented the ligand part of the complex, while the bluish colours indicated the areas of the proteins to which the ligands interacted.

The 3D visualization figures showed the top five interaction complexes for each receptor, and their binding energies. Orange coloured parts represent the ligand part of the interaction complex and blue regions indicate the areas of the proteins bound to the ligand (Figure 1, Figure 2, Figure 3, Figure 4, Figure 5).

## Ligand-protein contacts:

The ligand atom interactions with the protein residues were analysed. It was observed that amino acid residues Arg273 (A), Phe504 (A), Tyr50 (A), Tyr127 (A), Ile54 (A), Val343 (A), Phe274 (A), Trp271 (A), Asp269 (A), Asn149 (A) and Met270 (A) were involved in the protein-ligand contacts during the 100ns simulation of complex boswellic acid-1R42. Amino acids with hydrophobic interactions were more prominent. Likewise, in complex pimecrolimus-4G3D, only Leu455 (A), interacted hydrophobically with the protein during simulation interactions. The BMS_345541-6VXX complex had residues such as Ala1020 (A), Ala1020 (B), Ala1020 (C), Asn1023 (B), Glu1027 (A), Ala1016 (B), and Ile1013 (A) that interacted with the protein during the simulation trajectory. Hydrophobic interactions were more prominent here. The GYY_4137-6VW1 complex demonstrated residues such as Glu406 (B), Glu375 (B), His374 (B), Arg518 (B), Pro346 (B), Leu370 (B), Ser409 (B), Lys363 (B), and Phe274 (B) interacting with the protein. The Ser261 (A), Glu260 (A), Ala399 (A), Trp267 (A), Ala266 (A), Trp380 (A), and Trp453 (A) were ligand atoms that associated with the protein during simulation of the complex triamcinolone hexacetonide and 7MEQ. Only those interactions that occurred more than 10% of the simulation time in the selected simulation trajectory (0-100ns) were considered and shown (Figure 7).

## Ligand root mean square fluctuations (RMSF):

The RMSF data showed that the interaction complexes of Boswellic acid-1R42 and pimecrolimus-4G3D were having fluctuations from 3 to beyond 4 Å, the complex GYY_4137-6VW1 had an RMSF variation between 2-4 Å, while BMS_345541-6VXX demonstrated RMSF deviation between 1.5-4 Å. The Triamcinolone hexacetonide-7MEQ complex showed an RMSF that varies between 1-2 Å, pointing towards the fit of the ligand on the protein. The Figure 8 illustrates these RMSF fluctuations among various complexes. These results provided insights on how the ligand fragments interact with the protein and their entropic role in the event of binding. These fluctuations are commonly broken-down atom by atom corresponding to the ligand's 2D structures.

## Study of interaction mechanism via molecular dynamic simulations:

High performance molecular dynamic simulation studies for the top 5 docked complexes, one for each receptor was carried out using Desmond from Schroedinger, in the Maestro GUI. The results elucidated the stabilities of the bound interaction complexes, pointing towards their robustness when used in natural conditions. The following sections explain the various simulation outcomes for the top five interaction complexes.

## Root mean square deviations (RMSD):

A cumulative RMSD graph plotted based on the RMSD values obtained after simulation of the complexes showed that simulation at 100ns equilibrated towards the end of the simulation trajectory for every complex (Figure 3). The RMSD varied between 3.5 to 4.5 Å for complex boswellic acid-1R42, and equilibrated at 3.5 Å at the end of 100ns. This implied that the complex was highly stable because the RMSD value was less than 6 Å. Likewise, the RMSD for GYY_4137-6VW1 complex deviated between 12-16 Å and stabilized at 16 Å, implying that although the state reached an equilibrium, the complex underwent conformational changes as the RMSD value was found to be higher. Similarly, pimecrolimus-4G3D had an RMSD of 25 Å, with lot of variations between 10-45 Å, suggesting extreme conformational modifications during the 100ns simulations. This huge change in the conformation could be due to the disorder of the protein. However, the complex reaching equilibrium suggests the robustness of docking and complex stability. The RMSD fluctuated between 1.6-2.8 Å for complex triamcinolone hexacetonide-7MEQ, and stabilised at 2.4 Å, demonstrating that the interaction complex is extremely stable. The complex BMS_345541-6VXX had an RMSD fluctuation between 2.5-4.0 Å, and stabilized at 3.5 Å. This docked complex was also found to be highly stable without conformational changes induced, due to its RMSD being less than 6 Å. It is evident that protein RMSD of complex Pimecrolimus with 4G3D was found to be the highest among all other complexes (Figure 3), followed by GYY_4137 with 6VW1. The protein RMSD values for the remaining three complexes were around the same range (Table 3).

## Protein RMSF:

The peaks in the Figure 9 indicate the fluctuations that occurred in protein areas measurement during MDS analysis. The results show that for Boswellic acid-1R42, the protein RMSF deviated between 0.8-7 Å and tall peaks were noted at 4.8 Å, 6.4 Å and 7.2 Å. Likewise, for complex pimecrolimus-4G3D, protein RMSF ranged between 3-27 Å, with implications of high conformational changes in the protein. Protein RMSF for the complex GYY_4137-6VW1 demonstrated variations of the RMSF values between 1.5-14 Å, with tall peaks noted at 10.5 Å, 13.5 Å and 14 Å. The interaction complex BMS_345541-6VXX had protein RMSF varying between 0.8-7.2 Å, with lot of conformational changes occurring during the simulation exercise at 100ns. The Triamcinolone hexacetonide-7MEQ complex evidenced protein RMSF between 0.6-5.4 Å, with the tallest peak observed at 5.4 Å.

## Protein-ligand interactions:

The simulation outcomes show that protein-ligand contacts for complex boswellic acid-1R42 had many hydrogen bonds and hydrophobic interactions along with a few water bridges. The amino acid residues Asn149, Gly268 and Leu503 demonstrated maximum hydrogen bonds with higher interaction fraction while the remaining residues possessed hydrophobic bonds. The complex pimecrolimus-4G3D demonstrated prominent water bridges and a few hydrogen and hydrophobic interactions. No ionic bonds were noted. Likewise, at residue Glu1017 (A), the highest interaction fraction for hydrogen bond was noticed, while for complex BMS_354451-6VXX, several water bridges and a few hydrophobic contacts were recorded. The GYY_4137-6VW1 complex had the highest interaction fraction for ionic bonds at residue His374, with the presence of least number of hydrogen bonds. Some hydrophobic interactions and presence of water bridges were observed with the residues of the protein. Hydrogen, hydrophobic associations along with water bridges were notable in complex triamcinolone hexacetonide-7MEQ, with highest interaction fraction for hydrogen bonds observed at residues Trp267 and Ala399. No ionic interactions were found in this case (Figure 10). The stacked bar charts shown here, are normalized outputs over the course of the trajectory: for instance, an interaction fraction value of 0.7 suggests that 70% of the simulation time the specific interaction is maintained. Values over 1.0 are possible as some protein residue might have made multiple contacts of same subtype with the ligand.

## Timeline charts for protein-ligand contacts:

**Figure 6:**
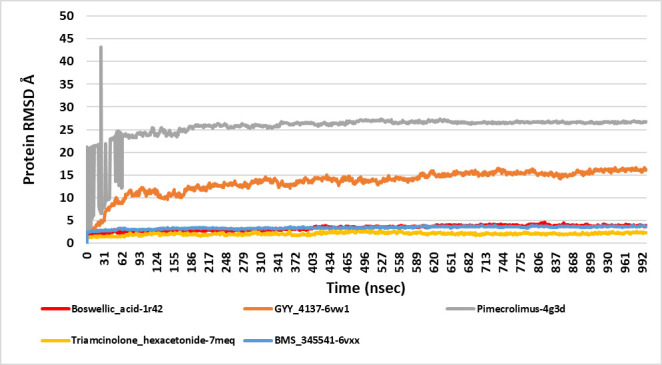
A cumulative protein RMSD graph for the five best docked complexes obtained via MDS. The y-axis showed the RMSD evolution of the proteins plotted against time in nano seconds. RMSD analysis indicated that the simulation had equilibrated towards the end. As observed from the graph, the RMSD after simulation showed stability towards the end. Changes between 1-3 Å are acceptable for smaller proteins, while changes in RMSD larger than that indicated that the proteins had undergone a large conformational change during MDS. It is essential that the RMSD values stabilized around a fixed value and the same was observed after MDS for all five complexes.

**Figure 7:**
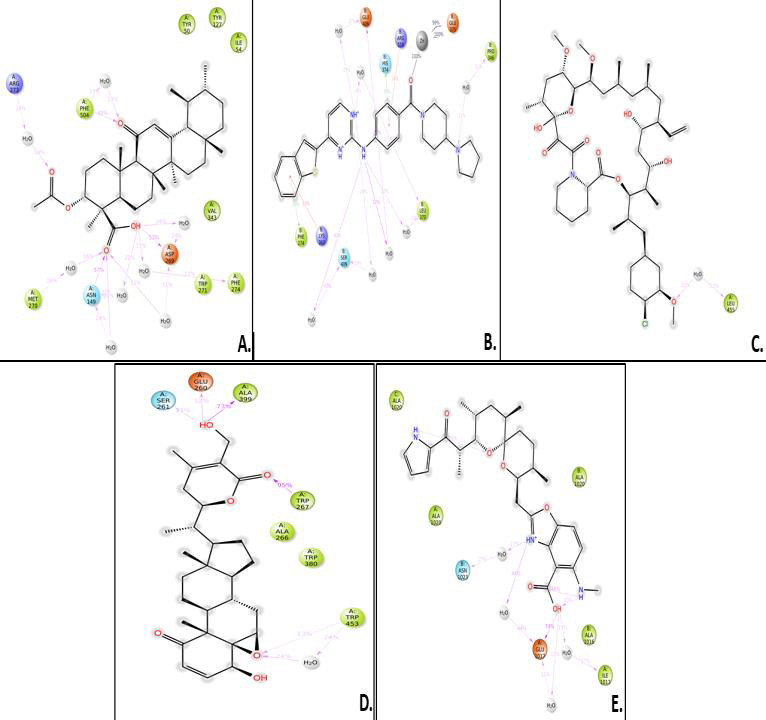
A cumulative figure showing the ligand-protein (LP) contacts for all the five docked complexes, obtained after MDS. A) Boswellic_acid-1R42, B) GYY_4137-6VW1, C) Pimecrolimus-4G3D, D) Triamcinolone_hexacetonide-7MEQ, E) BMS_345541-6VXX. The figure showed different types of amino acid contacts between the ligand and protein during MDS.

**Figure 8:**
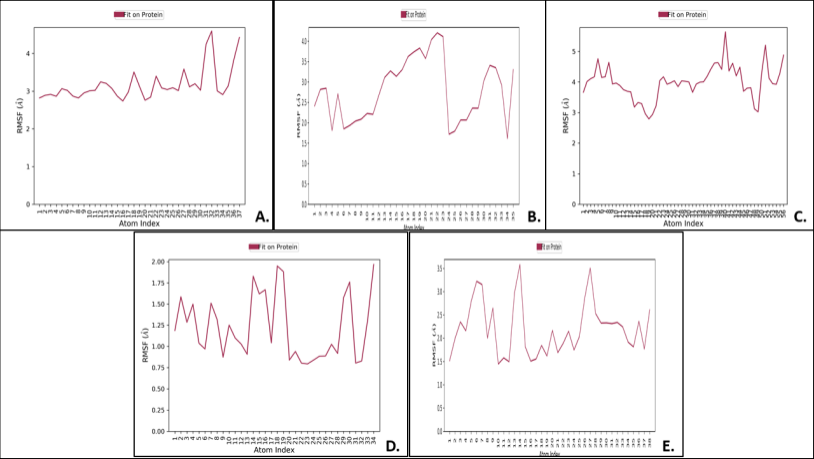
The ligand RMSF values for all five complexes which represents the ligand fit on the protein and shows the fluctuations of the ligand with respect to the proteins (displayed in violet colour). It represented how the ligands interacted with the proteins. The RMSF is generally measured on the heavy atoms of the ligand by first aligning the protein-ligand complex on the backbone of the protein and is useful in characterizing changes in the positions of the ligand atoms. A) Boswellic_acid-1R42; B) GYY_4137-6VW1; C) Pimecrolimus-4G3D, D) Triamcinolone_ hexacetonide-7MEQ, E) BMS_345541-6VXX complexes.

**Figure 9:**
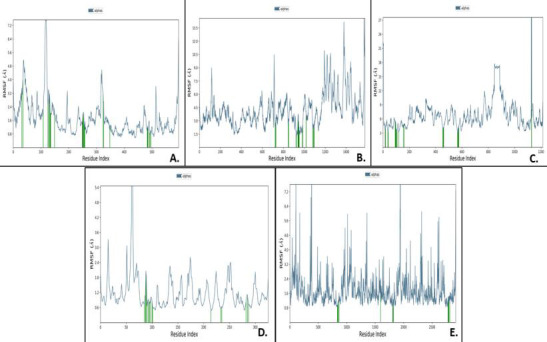
Graphs for protein RMSF with their ligand contacts for all top docked complexes, obtained as a result after MDS. A) Boswellic_acid-1r42, B) GYY_4137-6vw1, C) Pimecrolimus-4g3d, D) Triamcinolone_hexacetonide-7meq, E) BMS_345541-6vxx. The peaks indicate the areas of the protein which fluctuate most during MDS. Green coloured vertical bars represent the residues of the proteins that interact with the ligands.

**Figure 10:**
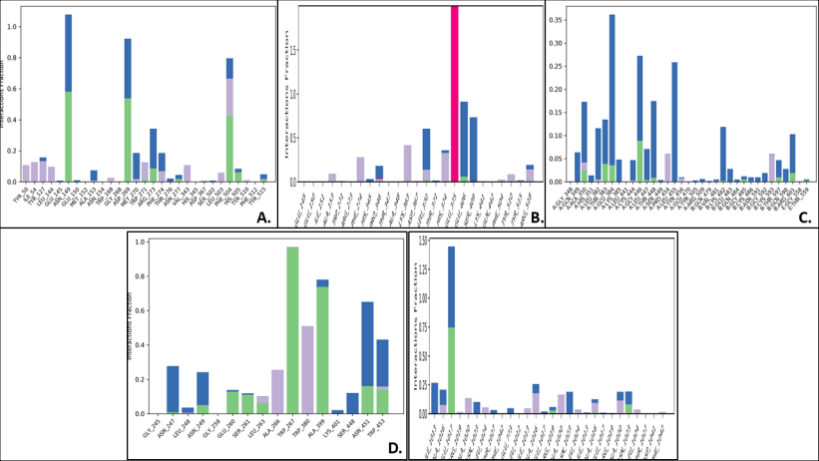
Graphs showing the interactions of the proteins with the ligand during MDS. Following molecules A) Boswellic_acid-1R42, B) GYY_4137-6VW1, C) Pimecrolimus-4G3D, D) Triamcinolone_hexacetonide-7MEQ, E) BMS_345541-6VXX. The y-axis represented the interaction fraction value and the x-axis represented the protein residues with which the ligands interacted. These interactions were categorized into 4 types- hydrophobic bonds, hydrogen bonds, ionic interactions and water bridges. The stacked bars were normalized over the trajectory course and values over 1.0 indicated that multiple contacts of the same sub-type with the ligands were possible.

To further assess the precise mechanism of the binding event through the simulation trajectory, the timeline charts for the same were studied for the protein-ligand contacts to gauge if the residues make single or multiple contacts. The top panel indicates the total number of specific contacts the protein makes with the ligand over the course of the simulation trajectory. The bottom panel indicates the residues that interact with the ligand in each frame of the trajectory . Some of these residues make more than one specific contact with the ligand in question and is represented by a darker shade of orange as per the scale provide to the plot. Boswellic acid-1R42 complex had residues Asn149 showing multiple ligand contacts between 0-100ns, while Asp269 exhibited multiple contacts between 20-100ns. Glu365 in complex GYY_4137-6VW1 demonstrated multiple contacts between 0-100ns. Similarly, Glu1017 (A) of complex BMS_354451-6VXX demonstrated multiple ligand contacts during the entire simulation trajectory between 0-100ns. Although not many multiple ligand contacts were noted in pimecrolimus-4G3D complex, Glu384 (A) exhibited more interactions between 0-70ns, Leu455 (A) had multiple contacts between 80-100ns of the trajectory. Maximum number of multiple contacts were reported for triamcinolone hexacetonide-7MEQ complex, with Trp267 exhibiting multiple associations between 0-100ns, Ala399 between 10-100 ns, followed by Asn451 and Trp453 between 0-100ns. These results point towards the mechanism between the drug-receptor complex that occurs during the binding event and contribute vastly towards the stability of the interaction complex as a result of which the binding efficacy maybe further explained under simulation conditions.Figure 6

## Ligand properties and secondary structure analysis:

The overall properties of the ligands such as RMSD, radius of gyration, intramolecular hydrogen bonds (IntraHB), molecular surface area (MolSA), solvent accessible surface area (SASA), and polar surface area (PSA) were analysed during the simulation at 100ns for top 5 docked complexes. The results indicated no existence of intra HBs for boswellic acid-1R42, GYY_4137-6VW1 and triamcinolone hexacetonide-7MEQ, while for pimecrolimus-4G3D and BMS_345541-6VXX, several intra HBs were identified between the 0-100ns trajectory. The ligand RMSD values, radius of gyration that measures the extendedness of the ligand, MolSA that calculates with the 1.4 Å probe radius and whose value is equivalent to the Vanderwaal's surface area and the PSA contributed by only the nitrogen and oxygen atoms in the ligand were also studied and illustrated. The complex boswellic_acid-1R42 was found to have more alpha helices, while the complex BMS_345541-6VXX was observed to have more beta strands during simulation.

## Discussion:

Over the past couple of years, drug repurposing for COVID-19 has taken on a much broader scope in research in-lieu of developing new drugs, due to time constraints and the urgent necessity of treatment options [33]. Therefore, an amalgamation of various translational bioinformatics approaches can allow swift drug repurposing applications on a much broader scale, aiding healthcare and management of the disease [34, 35]. The mechanism of SARS-CoV-2 infection from previous studies suggests it consisting of many complexes in coronavirus spike protein associated with ACE2 (angiotensin converting enzyme-2) and TMPRSS2 (transmembrane serine protease 2) receptors. The TMPRSS2 is the receptor-binding domain (RBD) of the spike that associates and binds to ACE2 protein's peptidase domain in humans [36]. In the present study, both these human receptors (1R42- Native Human Angiotensin Converting Enzyme-Related Carboxypeptidase and 7MEQ- Crystal structure of human TMPRSS2 in complex with Nafamostat) were considered to better comprehend the binding of the ligands to the host. Our results showed that binding capabilities of boswellic acid to human ACE2 and triamcinolone hexacetonide to TMPRSS2 were stable, indicating that even at molecular level, these two ligands play a major role in intervening the ACE2 and TMPRSS2 pathways. Additionally, the NF-κB pathway, activated by the cytokine storms in patients, has also been implicated in the extrapulmonary manifestations of COVID-19. A recent review of 22 observational studies, inclusive of about 17,391 patients suffering from COVID-19, described acute injury to the kidney at a rate of 11%, with 6.8% of them requiring renal replacement [37]. Therefore, targeting this pathway in humans via potential NF-κB inhibitors may help fight COVID-19. The present study showed that immunomodulation targetting NF-κB, by blocking the protein via pimecrolimus showed complex stability towards the end of the 100ns simulation, with conformational changes induced, pointing towards a potential NF-κB inhibition in COVID-19 surge. Studies report that inhibitors of NF-κB reduce the cytokine storms, assuaging the severity of the disease and its effects [38]. Reports state that drugs such as Cromolyn can be repurposed against NF-κB-based targets [39]. However, the results from our studies, point towards potential NF-κB pathway inhibitors, having their roles very significant considering their unique benefits [40]. A recent study has also implicated boswellic acid as a potential therapeutic agent against COVID-19 amongst the elderly. The study stated moderate use of boswellic acid to enhance the adaptive immune system and also suggests the role of boswellic acid in the suppression of uncontrolled activation of innate immunity, in cases of infection, as in the case of cytokine storms [41]. This further corroborates the results obtained from our studies. Likewise, other previous studies have been accounted, such as GYY_4137, a slow H2S-releasing compound, a potent antiviral and anti-inflammatory molecule that can be used against COVID-19 and BMS_345541 as a potential anti-viral compound [41]. There are some interesting reports on the availability of other small molecules such as Tamaridone and Hyoscyamine that have previously worked well against SARS-CoV-2 spike glycoprotein, Scutifoliamide-A and Rotiorinol-C against replicase polyprotein of the virus, which were identified from in-silico docking and simulation studies. The results presented in the current work are novel in the selection of the receptor and the drug leads that were employed in the current strategy that formed stable complexes at 100ns of simulation studies in a biological environment and hence recommended for further clinical trials.

## Conclusion:

Data sheds light on targeting of NF-κB human (host) transcription factor that can reduce the effect of SARS-CoV-2 viral attack, and also provides molecular insights into the action of various currently studied drugs on the above chosen receptor that can open up vistas for potential therapeutics discovery against COVID-19.

## Author contributions:

VM conceived the idea and study design, PB, ASS, CK, VN, NHK, SB, SMT and AR carried out the data analyses and PB, VN, JK and VM drafted the manuscript and VM provided comments and performed corrections. All authors read and approved the final manuscript.

## Funding Declaration:

The authors acknowledge the Bangalore Bio Innovation Centre, Karnataka, Department of Electronics, IT, BT and S and T, Government of Karnataka, India, for funding acquisition towards paying the publication cost.

## Data availability statement:

Not applicable.

## Figures and Tables

**Table 1:** Top five shortlisted docked complexes for each receptor with the best binding energies

**S. No**	**Receptor**	**Docked complex**	**Energy (kcal/mol)**
1	1R42	1r42_Boswellic acid	-9.2
		1r42_Pimecrolimus	-9
		1r42_Tacrolimus	-9
		1r42_IKK-16	-8.9
		1r42_Triamcinolone_hexacetonide	-8.7
2	4G3D	4g3d_Pimecrolimus	-9.1
		4g3d_Tacrolimus	-9.1
		4g3d_GYY_4137	-8.9
		4g3d_IKK-16	-8.9
		4g3d_Boswellic_acid	-8.6
3	6VW1	6vw1_GYY_4137	-10.3
		6vw1_Triamcinolone_acetonide	-10.2
		6vw1_Triamcinolone_hexacetonide	-9.9
		6vw1_Sulindac	-9.5
		6vw1_Diacerein	-9.4
4	6VXX	6vxx_BMS_345541	-10.1
		6vxx_GYY_4137	-9.9
		6vxx_Boswellic_acid	-9.7
		6vxx_IKK-16	-9.7
		6vxx_Triamcinolone_acetonide	-9.6
5	7MEQ	7meq_Triamcinolone_hexacetonide	-8.7
		7meq_GYY_4137	-8.7
		7meq_Mesalamine	-8.2
		7meq_Resveratrol	-8
		7meq_Lactacystin	-7.8
		7meq_sulindac	-7.8

**Table 2:** The lists of amino acid interactions between the docked protein and ligand complexes for the shortlisted top molecules for each receptor, as obtained from PLIP tool

**Sl.no**	**Receptor**	**Docked complex**	**Amino acid residues with hydrogen bond interactions**	**Amino acid residues with hydrophobic interactions**
1		1r42_Boswellic acid	Arg273 (A), Asn277 (A)	Trp271 (A), Phe274 (A), Phe504 (A)
2		1r42_Pimecrolimus	Ala348 (A)	Ala348 (A), His378 (A)
3	1R42	1r42_Tacrolimus	Ala348 (A)	Phe40 (A), Ala348 (A), Trp349 (A), His378 (A)
4		1r42_IKK-16	Asp350 (A), Asp382 (A)	Phe40 (A), Asp350 (A), Phe390 (A)
5		1r42_Triamcinolone_hexacetonide	Asn103 (A), Arg393 (A), Ser77 (A), Gln102, (A), Ser106 (A)	Leu73 (A), Leu100 (A), Phe390 (A)
6		4g3d_Pimecrolimus	Ala350 (A), Thr383 (A), Glu384 (A)	Pro454 (A), Tyr456 (A), Lys482 (B), Thr597 (B)
7	4G3D	4g3d_Tacrolimus	Lys576 (D), Gln631 (D), Arg510 (D), Asp574(D)	Asp574 (D), Ile639 (D)
8		4g3d_GYY_4137	Gln628 (E)	Leu541 (D), Ala627 (E), Ala575 (D), Arg510 (D), Ala575 (D), Ala627, (E), Pro624 (E), Ile618 (E)
9		4g3d_IKK-16	Arg408 (E), Ser476 (E)	Val414 (E), Asp519 (E), Leu522 (E)
10		4g3d_Boswellic_acid	Gln349 (A), Ala350 (A), Thr448 (A), His594(B)	Leu382 (A), Thr448 (A), Pro454 (A), Thr597 (B)
11		6vw1_GYY_4137	Glu406 (B), His345 (B)	Phe274 (B), Thr371 (B), Phe504 (B)
12		6vw1_Triamcinolone_acetonide	Glu406 (B), Arg 518 (B), Glu402 (B)	Phe274 (B), Phe504 (B), Tyr510 (B), Tyr515 (B)
13	6VW1	6vw1_Triamcinolone_hexacetonide	Tyr515 (B), Thr276 (B)	Phe274 (B), Thr276 (B), Thr445 (B)
14		6vw1_Sulindac	Glu375 (B), Glu406 (B), Arg518 (B)	Phe274 (B), Pro346 (B), Leu370 (B), Tyr515 (B)
15		6vw1_Diacerein	Arg518 (B), Glu406 (B), Ser409 (B), Phe274(B), Asn277 (B)	Phe274 (B), Asp367 (B), Thr445 (B)
16		6vxx_BMS_345541	Asn1023 (B), Arg1019 (B)	Ala1020 (B), Ala1020 (C), Leu1024 (A), Leu1024 (C)
17		6vxx_GYY_4137	Thr768 (B)	Thr302 (A), Tyr313 (A), Thr768 (B)
18	6VXX	6vxx_Boswellic_acid	Arg983 (B), Leu517 (A), Leu518 (A)	Asp198 (B), Tyr200 (B), Phe464 (A), Glu516 (A), Leu518 (A)
19		6vxx_IKK-16	Gln1036 (A)	Trp886 (A), Gln1036 (A), Lys1038 (A), Val1040 (C), Tyr1047 (C)
20		6vxx_Triamcinolone_acetonide	Thr1027 (C), Lys1028 (B), Glu725 (B)	Gln784 (C), Leu1024 (B), Ala1026 (C), Thr1027 (C), Phe1042 (B)
21		7meq_Triamcinolone_hexacetonide	Asp451 (A), Ser448 (A), Glu260 (A), Trp267(A), Leu263 (A)	Glu260 (A), Trp267 (A), Trp380 (A), Trp453 (A)
22		7meq_GYY_4137	Asn304 (A), Asn303 (A), Ser333 (A)	Lys300 (A), Asn303 (A), Lys330 (A), Val331 (A), Ile332 (A)
23	7MEQ	7meq_Mesalamine	Asn249 (A), Leu248 (A), Asp247 (A), Trp453(A) (A)	Leu248 (A), Glu260 (A), Leu263 (A), Ala266 (A), Ala399 (A), Trp453
24		7meq_Resveratrol	Trp306 (A), Gln276 (A), Asn277 (A), His274(A), Thr309 (A)	Phe311 (A), Tyr322 (A)
25		7meq_Lactacystin	Gln276 (A), Asn277 (A)	Phe311 (A), Tyr322 (A)
26		7meq_sulindac	Phe194 (A), Asn192 (A), Asp359 (A)	Ile242 (A), Pro288 (A), Lys362 (A)

**Table 3:** Docked complexes, their average RMSD values and RMSF values of the proteins after MDS analysis.

**S. No.**	**Docked complex**	**RMSD of protein**	**RMSF of protein**
1	Boswellic_acid-1r42	3.340717	1.7238
2	GYY_4137-6vw1	13.50393	4.0418
3	Pimecrolimus-4g3d	25.49716	6.8255
4	Triamcinolone_hexaacetonide-7meq	2.050429	1.1875
5	BMS_345541-6vxx	3.37546	1.7492
